# The SP4–NUPR1–SELM Axis Promotes Glycolytic Reprogramming and Stemness-Associated Phenotypes in Bladder Cancer

**DOI:** 10.3390/cancers18203396

**Published:** 2026-10-20

**Authors:** Hannuo Deng, Rui Sun, Guangming He, Yuchen Tan, Lei Zhang, Shenglin Gao, Yichao Zhu, Lifeng Zhang, Li Zuo, Zhiqi Tang

**Affiliations:** 1 Department of Urology & Urology Laboratory, The Third Affiliated Hospital of Nanjing Medical University, Changzhou 213003, China; denghannuo@stu.njmu.edu.cn (H.D.);; 2 Department of Physiology, School of Basic Medical Sciences, Nanjing Medical University, Nanjing, 211166, China; 3 Department of Radiology, The Third Affiliated Hospital of Nanjing Medical University, Changzhou 213003, China

**Keywords:** bladder cancer, SP4, NUPR1, SELM, glycolysis, stemness, metabolic reprogramming, tumor progression

## Abstract

**Background:** Metabolic adaptation and stemness-associated programs contribute to bladder cancer growth. This study investigated the regulatory and functional relationships among specificity protein 4 (SP4), nuclear protein 1 (NUPR1), and selenoprotein M (SELM). **Methods:** TCGA-BLCA analyses were combined with silencing, overexpression, and rescue experiments in 5637 and T24 cells. Viability, migration, invasion, colony formation, glucose uptake, lactate production, extracellular acidification, oxygen consumption, and OCT4/SOX2 expression were assessed. Protein interactions and promoter-associated regulation were examined using co-immunoprecipitation, immunofluorescence, chromatin immunoprecipitation, and reporter assays. Xenograft experiments in female BALB/c nude mice evaluated NUPR1 depletion with or without SP4 overexpression. **Results:** Higher NUPR1 expression was associated with higher glycolysis- and stemness-associated transcriptional scores in TCGA-BLCA tumors. NUPR1 depletion reduced viability, glycolytic activity, glucose uptake, lactate production, and OCT4/SOX2 expression while increasing oxygen consumption. SP4 interacted with NUPR1 and showed enrichment at the NUPR1 promoter region. NUPR1 promoted SELM expression and reporter activity, with enrichment detected at the SELM promoter region. SP4 or SELM overexpression partially rescued the effects of NUPR1 depletion. SELM silencing attenuated NUPR1-associated increases in viability, migration, invasion, and colony formation. NUPR1 depletion suppressed xenograft growth, which was partially rescued by SP4 overexpression. **Conclusions:** These findings support functional relationships among SP4, NUPR1, and SELM that connect glycolytic activity with stemness-associated phenotypes in bladder cancer models. The results provide a basis for further investigation of this regulatory network in cancer metabolism.

## Simple Summary

Bladder cancer cells adapt their energy use to support growth and survival. This study investigated how three proteins, specificity protein 4, nuclear protein 1, and selenoprotein M, contribute to these processes. Public tumor data, experiments in bladder cancer cells, and a mouse tumor model linked nuclear protein 1 to glucose metabolism and molecular features associated with stem-like cellular states. Reducing nuclear protein 1 decreased these features and slowed tumor growth. Increasing specificity protein 4 or selenoprotein M partly reversed the changes observed in cultured cells. The findings identify a regulatory network connecting glucose metabolism with tumor-associated cellular behavior and provide a basis for further investigation of this network in bladder cancer.

## 1. Introduction

Bladder cancer imposes a substantial disease burden because of its incidence, repeated treatment requirements, and variable clinical course [1]. Management and prognosis differ between non-muscle-invasive and muscle-invasive disease. For non-muscle-invasive bladder cancer (NMIBC), recurrence and the risk of subsequent progression necessitate continued surveillance and risk-adapted treatment [2,3,4,5]. Muscle-invasive bladder cancer (MIBC) requires more intensive management and remains associated with adverse outcomes despite available therapies [5,6]. These clinical challenges motivate investigation of the cellular processes that support bladder cancer growth and adaptation.

Metabolic reprogramming is a fundamental hallmark of cancer. Since Warburg first proposed that cancer cells preferentially utilize glycolysis even in the presence of sufficient oxygen, accumulating evidence has shown that glycolytic remodeling provides not only rapid adenosine triphosphate (ATP) generation but also metabolic intermediates required for biomass synthesis and redox homeostasis [7,8,9,10,11,12]. Importantly, cancer metabolism is now understood as a dynamic and adaptive network in which glycolysis and oxidative phosphorylation can be rebalanced according to microenvironmental stress and treatment pressure to support tumor growth, invasion, and survival [9,10,11,12,13]. Therefore, identifying key molecules that connect stress adaptation with metabolic remodeling may provide new mechanistic insights and therapeutic opportunities.

Cancer stemness and cellular plasticity represent another major determinant of tumor recurrence and progression. Cancer stem-like cells possess enhanced self-renewal capacity, tumor-initiating potential, and resistance to conventional therapies, thereby contributing to recurrence, metastasis, and treatment failure [14,15,16,17]. Stemness-associated transcription factors such as octamer-binding transcription factor 4 (OCT4) and SRY-box transcription factor 2 (SOX2) are closely linked to maintenance of the undifferentiated state and aggressive behavior across multiple cancer types [18,19]. Emerging evidence also suggests that metabolic programs and stemness are tightly interconnected, as specific metabolic states can sustain stemness-related transcriptional networks and facilitate phenotypic plasticity [9,12,20].

Nuclear protein 1 (NUPR1), also known as p8, is a stress-inducible nuclear protein implicated in multiple cellular processes, including transcriptional regulation, apoptosis, autophagy, cell cycle progression, and redox homeostasis [21,22,23,24]. Increasing evidence indicates that NUPR1 enables cancer cells to adapt to environmental and metabolic stress, thereby promoting tumor cell survival and therapeutic resistance [22,23,24,25,26]. Moreover, pharmacological inhibition of NUPR1 has shown anticancer potential, highlighting its translational relevance [26]. However, how NUPR1 connects stress-responsive regulation with glucose metabolism and stemness-associated programs in bladder cancer remains incompletely understood.

Members of the specificity protein (SP) family of transcription factors, including specificity protein 1 (SP1) and specificity protein 4 (SP4), are known to regulate multiple cancer-related biological processes, such as proliferation, invasion, and metabolic adaptation [27,28,29,30,31]. In parallel, selenium and selenoproteins participate in redox control and stress adaptation, and dysregulation of this system has been implicated in tumor development and progression [32,33,34,35,36]. Selenoprotein M (SELM), a member of the selenoprotein family, has been suggested to participate in intracellular homeostasis, although its role in bladder cancer remains poorly defined.

Here, we combined NUPR1-stratified TCGA-BLCA analyses with perturbation experiments in 5637 and T24 cells to investigate relationships among transcription-associated regulation, glucose metabolism, and stemness-associated cellular features. We examined SP4–NUPR1 association and promoter-related regulation, followed by complementary experiments testing the contribution of SELM to NUPR1-associated phenotypes. Xenograft experiments further evaluated the effects of NUPR1 depletion and SP4 overexpression on tumor growth. This approach was designed to characterize functional relationships within the tested models.

## 2. Materials and Methods

### 2.1. Bioinformatics Analysis

TCGA-BLCA transcriptomic data and corresponding sample annotations were obtained from the UCSC Xena platform [37]. The dataset included 409 primary tumor samples from 406 patients and 19 normal samples. Normal samples were included in the initial preprocessing, whereas NUPR1-stratified analyses were restricted to primary tumor samples. Gene identifiers were mapped using GENCODE v36. When multiple entries mapped to the same gene symbol, the entry with the highest mean expression was retained. Bioinformatics analyses were performed using R (version 4.5.1). The expression matrix was back-transformed and rounded before TMM normalization using edgeR (version 4.6.3), followed by differential expression analysis using the limma-voom workflow implemented in limma (version 3.64.3). Tumor samples were divided into NUPR1-high and NUPR1-low groups according to median NUPR1 expression, yielding 205 and 204 samples, respectively. Genes with an FDR < 0.05 and an absolute log2 fold change > 0.5 were considered differentially expressed. Gene sets were obtained from the Molecular Signatures Database (MSigDB, version 2026.1.Hs). GSEA was performed using clusterProfiler (version 4.18.4), with genes ranked by the moderated t-statistic from the limma differential expression analysis. Positive ranking values indicated higher expression in the NUPR1-high group. Single-sample gene set enrichment analysis (ssGSEA) was performed using GSVA (version 2.2.0) to calculate glycolysis- and stemness-associated transcriptional scores based on HALLMARK_GLYCOLYSIS from the Hallmark collection and BENPORATH_NOS_TARGETS from the C2:CGP collection, respectively. The latter represents a transcriptional signature associated with NANOG, OCT4, and SOX2 targets in human embryonic stem cells and was used to assess stemness-associated transcriptional programs. Associations between NUPR1 expression, transcriptional scores, and candidate genes were assessed using Spearman correlation analysis. Selenoprotein and SP-family genes were examined to nominate candidates for subsequent experiments.

### 2.2. Cell Culture and Transfection

Human bladder cancer cell lines 5637 and T24 were obtained from the Cell Bank of the Chinese Academy of Sciences (Shanghai, China) and maintained in DMEM (cat. no. 41402ES76; YEASEN Biotechnology, Shanghai, China) supplemented with 10% fetal bovine serum (cat. no. 40130ES76; YEASEN Biotechnology) and 1% penicillin–streptomycin (cat. no. 60162ES76; YEASEN Biotechnology) at 37 °C in a humidified atmosphere containing 5% CO_2_. STR authentication and mycoplasma testing were performed by the supplier before delivery, and the corresponding quality-control reports are retained. siRNAs targeting NUPR1 or SELM, negative-control siRNA, and overexpression constructs were obtained from GenePharma (Shanghai, China). One siRNA duplex targeting NUPR1 and one targeting SELM were used in the functional experiments. Their sequences and the negative-control sequence are listed in Supplementary Table S2.

NUPR1, SP4, and SELM overexpression constructs were generated using the pcDNA3.1(+) backbone. N-terminal HA-tagged NUPR1 and N-terminal Flag-tagged SP4 constructs were used for exogenous co-immunoprecipitation. Transient transfection was performed using Lipofectamine 3000 Transfection Reagent (cat. no. L3000001; Invitrogen, Thermo Fisher Scientific, Carlsbad, CA, USA). Knockdown and overexpression were assessed 48 h after transfection, and metabolic assays were performed 72 h after transfection. SP4 or SELM was overexpressed in NUPR1-depleted cells for rescue experiments, while SELM was depleted in NUPR1-overexpressing cells for complementary functional experiments. Stable 5637 cells for xenograft experiments were generated by lentiviral transduction.

### 2.3. RT-qPCR and Western Blotting

Total RNA was extracted using an RNA isolation reagent from Aidlab Biotechnologies (Beijing, China) and reverse-transcribed using the HiScript II 1st Strand cDNA Synthesis Kit (+gDNA wiper) (cat. no. R212-01; Vazyme, Nanjing, China). RT-qPCR was performed using AceQ Universal SYBR qPCR Master Mix (cat. no. Q511-03; Vazyme) on a PCR-9601-NS real-time PCR instrument (RocGene, Beijing, China). Gene-specific primers were synthesized by GenScript (Nanjing, China), and their sequences are listed in Supplementary Table S1. NUPR1, SP4, and SELM expression was normalized to GAPDH, and relative expression was calculated using the 2^−ΔΔCt^ method.

Total protein was extracted using RIPA lysis buffer (cat. no. G2002; Servicebio, Wuhan, China) supplemented with a protease inhibitor cocktail (cat. no. G2006; Servicebio), PMSF (cat. no. G2008; Servicebio), and a phosphatase inhibitor (cat. no. G2007; Servicebio). Twenty micrograms of total protein per lane were separated by SDS-PAGE and transferred to PVDF membranes (cat. no. BSP0161; Pall Corporation, Port Washington, NY, USA), followed by blocking with 5% non-fat milk. Membranes were incubated overnight at 4 °C with primary antibodies against OCT4 (cat. no. ab200834), SOX2 (cat. no. ab92494), SP1 (cat. no. ab231778), SP4 (cat. no. ab151777), NUPR1 (cat. no. ab234696), SELM (cat. no. ab133681), and GAPDH (cat. no. ab8245), all from Abcam (Cambridge, UK), or ZBTB7B (cat. no. 11341-1-AP; Proteintech, Rosemont, IL, USA). After washing, membranes were incubated with HRP-conjugated anti-rabbit IgG (cat. no. AS014) or anti-mouse IgG (cat. no. AS003) secondary antibodies from ABclonal (Wuhan, China), as appropriate. Signals were detected using an enhanced chemiluminescence reagent (cat. no. P0018M; Beyotime Biotechnology, Shanghai, China). Xenograft tissues were processed using the same immunoblotting procedure. Western blot source images are provided in Supplementary Figure S1.

### 2.4. Cell Viability, Glycolysis, and Mitochondrial Respiration Assays

Cell viability was assessed using a CCK-8 assay kit (cat. no. HY-K0301; MedChemExpress, Monmouth Junction, NJ, USA). Cells were seeded in 96-well plates, incubated with CCK-8 reagent, and assessed by absorbance measurement at 450 nm using a Multiskan MK3 microplate reader (Thermo Fisher Scientific, Vantaa, Finland). Extracellular acidification rate (ECAR) and oxygen consumption rate (OCR) were measured using a Seahorse XFe24 Extracellular Flux Analyzer (Agilent Technologies, Santa Clara, CA, USA). Seahorse XF DMEM medium (cat. no. 103575-100; Agilent Technologies) was used for the assays. For the glycolysis stress test, glucose (cat. no. G7021), oligomycin (cat. no. 75351), and 2-deoxy-D-glucose (cat. no. D8375) were injected sequentially. For the mitochondrial stress test, oligomycin, FCCP (cat. no. C2920), and rotenone/antimycin A (cat. nos. R8875 and A8674, respectively) were injected sequentially. These compounds were obtained from Sigma-Aldrich (St. Louis, MO, USA). ECAR and OCR measurements were normalized to the cell number determined after the assay. Data were analyzed using Seahorse Wave Desktop software (version 2.6.4; Agilent Technologies, Santa Clara, CA, USA).

Lactate production and glucose uptake were measured using a lactate assay kit (cat. no. ml650233) and a glucose uptake assay kit (cat. no. ml076790), respectively, both from Shanghai Enzyme-linked Biotechnology Co., Ltd. (Shanghai, China). Lactate production and glucose uptake were normalized to the number of cells in each sample.

### 2.5. Immunofluorescence and Co-Immunoprecipitation

For immunofluorescence, cells grown on coverslips were fixed, permeabilized, and blocked before incubation with a rabbit anti-NUPR1 antibody (cat. no. ab234696; Abcam, Cambridge, UK) and a mouse anti-SP4 antibody (clone B-1; cat. no. sc-390124; Santa Cruz Biotechnology, Dallas, TX, USA). NUPR1 and SP4 were visualized using Alexa Fluor 488-conjugated goat anti-rabbit IgG (cat. no. A-11034) and Alexa Fluor 594-conjugated goat anti-mouse IgG (cat. no. A-11032), respectively, both from Invitrogen, Thermo Fisher Scientific (Eugene, OR, USA). Nuclei were counterstained with DAPI (cat. no. C1002; Beyotime Biotechnology, Shanghai, China). Images were acquired using an IX51 fluorescence microscope (Olympus, Tokyo, Japan), with identical exposure settings for the corresponding experimental comparisons.

For co-immunoprecipitation, cells were lysed in IP lysis buffer (cat. no. G2038; Servicebio, Wuhan, China). Lysates were incubated with the indicated antibodies or rabbit control IgG (cat. no. AC005; ABclonal, Wuhan, China), followed by Protein G Plus/Protein A agarose (cat. no. IP05; Millipore, Burlington, MA, USA). Immunoprecipitates were washed, eluted, and analyzed by Western blotting. Exogenous co-immunoprecipitation was performed after co-transfection with N-terminal HA-tagged NUPR1 and N-terminal Flag-tagged SP4 constructs. HA-tagged and Flag-tagged proteins were detected using an anti-HA antibody (clone C29F4; cat. no. 3724; Cell Signaling Technology, Danvers, MA, USA) and an anti-Flag antibody (clone M2; cat. no. F1804; Sigma-Aldrich, St. Louis, MO, USA), respectively.

### 2.6. ChIP-qPCR and Dual-Luciferase Reporter Assay

ChIP-qPCR was performed using primers targeting regions of the NUPR1 and SELM promoters to assess SP4 and NUPR1 enrichment, respectively. Cells were cross-linked, lysed, and sonicated using a JY92-IIN ultrasonic processor (Scientz, Ningbo, China) and chromatin was immunoprecipitated using anti-SP4, anti-NUPR1, or control IgG antibodies. Purified DNA was analyzed using 2× SYBR Green qPCR Master Mix (High ROX) (cat. no. G3322; Servicebio, Wuhan, China) on a StepOnePlus Real-Time PCR System (Applied Biosystems, Thermo Fisher Scientific, Foster City, CA, USA), and enrichment was normalized to input.

Approximately 2-kb NUPR1 and SELM promoter fragments were used in the reporter assays. The mutant NUPR1 reporter contained substitutions within the candidate SP4-binding motif, while the remaining promoter sequence was unchanged. Cells were co-transfected with the corresponding reporter constructs and the indicated siRNAs or expression plasmids. Luciferase activity was measured 24–48 h after transfection using the Dual-Luciferase Reporter Assay System (cat. no. E1910; Promega, Madison, WI, USA). Firefly luciferase activity was normalized to Renilla luciferase activity.

### 2.7. Migration, Invasion, and Colony Formation Assays

Migration and invasion were assessed using Transwell chambers with 8.0-μm pore polycarbonate membranes (cat. no. 3422; Corning, Corning, NY, USA) without or with Matrigel Matrix coating (cat. no. 356234; Corning), respectively. Cells were suspended in serum-free medium and placed in the upper chamber, while complete medium was added to the lower chamber. Migrated or invaded cells were fixed with 4% paraformaldehyde (cat. no. BL539A; Biosharp, Hefei, China), stained with 0.1% crystal violet (cat. no. 60506ES60; YEASEN Biotechnology, Shanghai, China), and imaged using an NIB410-FL inverted microscope (Nexcope, Ningbo, China) before quantification. For colony formation assays, transfected cells were seeded at 500 cells per well in six-well plates and cultured for 10–14 days. Colonies were fixed with 4% paraformaldehyde (cat. no. BL539A; Biosharp, Hefei, China), stained with 0.1% crystal violet (cat. no. 60506ES60; YEASEN Biotechnology, Shanghai, China), photographed, and counted.

### 2.8. Xenograft Assay and Immunohistochemistry

Animal experiments were approved by the Institutional Animal Care and Use Committee and the Ethics Review Board of Nanjing Medical University (JCDW-2021-019; 16 March 2021). Female BALB/c nude mice aged 5–6 weeks were obtained from Beijing Maidikangna Biotechnology (Beijing, China) and maintained under specific-pathogen-free conditions. Twenty-four mice were randomly allocated using computer-generated random numbers to four groups (*n* = 6/group): sh-NC, sh-NUPR1, sh-NUPR1+Vector, and sh-NUPR1+oe-SP4.

Stable 5637 cells (5 × 10^6^ cells in 100 μL of a 1:1 mixture of PBS and Matrigel Matrix [cat. no. 356234; Corning, Corning, NY, USA]) were injected subcutaneously into the flank under brief isoflurane anesthesia. Tumor dimensions were measured every 5–7 days, and volume was calculated as length × width^2^/2. Mice were euthanized on day 28, and tumors were collected for weighing, Western blotting, and immunohistochemistry. Tumor measurement, histological scoring, and data analysis were performed blinded to group allocation. Pre-injection illness and failed engraftment were prespecified exclusion criteria; no animals or data points were excluded. Animal health was monitored daily, with humane endpoints including >15% body-weight loss, tumor ulceration, impaired activity, or tumor volume > 1500 mm^3^.

For immunohistochemistry, paraffin-embedded tumor sections were deparaffinized, rehydrated, subjected to antigen retrieval, and incubated with primary antibodies against NUPR1 (cat. no. ab234696), SELM (cat. no. ab133681), or SP4 (cat. no. ab151777), all from Abcam (Cambridge, UK). Signals were detected using an HRP-conjugated goat anti-rabbit IgG secondary antibody (cat. no. AS014; ABclonal, Wuhan, China) and a DAB chromogenic reagent (cat. no. G1212; Servicebio, Wuhan, China). Images were acquired using an E100 microscope (Nikon, Tokyo, Japan).

### 2.9. Statistical Analysis

In vitro experiments were independently repeated at least three times, with the number of independent experiments specified in the corresponding figure legends. Xenograft experiments included six mice per group. Quantitative data are presented as mean ± standard deviation. Statistical analyses were conducted using GraphPad Prism (version 10; GraphPad Software, Boston, MA, USA) and R (version 4.5.1; R Foundation for Statistical Computing, Vienna, Austria). Two-group comparisons of experimental measurements were analyzed using two-sided unpaired Student’s t-tests. Differences in ssGSEA scores between the NUPR1-high and NUPR1-low tumor groups were assessed using the Wilcoxon rank-sum test. Comparisons among multiple groups were analyzed using one-way ANOVA followed by Tukey’s multiple-comparison test. Tumor growth curves were presented to illustrate changes over time, whereas statistical comparisons of tumor volume were performed at the day-28 endpoint. CCK-8 time-course data were presented as viability profiles, with between-group statistical comparisons performed at the final assay time point. Normality was assessed using the Shapiro–Wilk test, and equality of variances was assessed using the Brown–Forsythe test. Spearman correlation analysis was used for gene-expression and transcriptional-score associations. Statistical comparisons are identified in the corresponding figure panels. A *p* value < 0.05 was considered statistically significant.

## 3. Results

### 3.1. NUPR1-Stratified Transcriptomic Analyses Identify Glycolysis- and Stemness-Associated Programs and Candidate Regulatory Partners

**Figure 1:**
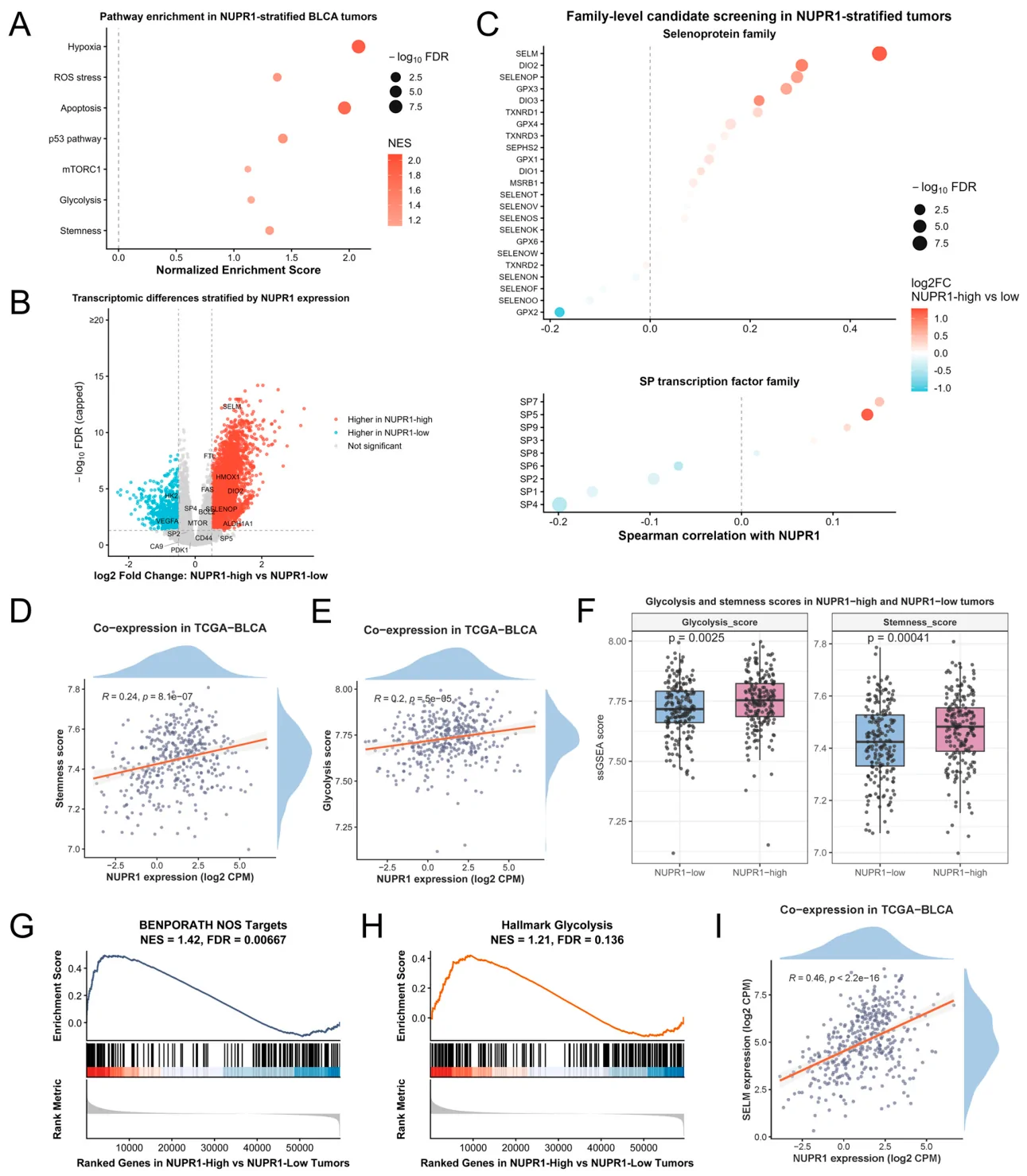
NUPR1-stratified transcriptome analyses in TCGA-BLCA tumors. Analyses included 409 primary tumor samples, divided into NUPR1-high (*n* = 205) and NUPR1-low (*n* = 204) groups. (**A**) Pathway enrichment analysis of NUPR1-associated transcriptional programs. (**B**) Volcano plot of differentially expressed genes between the two groups. Selected metabolism-, stemness-, stress-response-, selenoprotein-, and SP-family-related genes are labelled. (**C**) Family-level screening of selenoprotein and SP-family genes. (**D**,**E**) Spearman correlations between NUPR1 expression and ssGSEA scores calculated using BENPORATH_NOS_TARGETS and HALLMARK_GLYCOLYSIS, respectively. (**F**) Comparison of the corresponding transcriptional scores between groups. (**G**,**H**) GSEA of BENPORATH_NOS_TARGETS and HALLMARK_GLYCOLYSIS. (**I**) Spearman correlation between NUPR1 and SELM expression. Statistical results are indicated in the corresponding panels.

To investigate NUPR1-associated transcriptional programs in bladder cancer, 409 TCGA-BLCA primary tumor samples were divided into NUPR1-high and NUPR1-low groups using median NUPR1 expression. Pathway analyses linked higher NUPR1 expression to glycolysis and several stress-responsive programs, including hypoxia, reactive oxygen species responses, apoptosis, p53 signaling, and mTORC1 signaling (Figure 1A).

Differential expression analysis identified broad transcriptomic differences between the two groups. Representative genes associated with glucose metabolism, stemness-related programs, stress responses, selenoproteins, and SP-family regulation were highlighted, including SELM, HMOX1, DIO2, ALDH1A1, HK2, VEGFA, and SP4 (Figure 1B). Family-level screening further nominated SELM and SP4 for subsequent experimental investigation (Figure 1C).

ssGSEA scores calculated using BENPORATH_NOS_TARGETS and HALLMARK_GLYCOLYSIS were positively associated with NUPR1 expression (Figure 1D,E), and both scores were higher in NUPR1-high tumors (Figure 1F). GSEA showed enrichment of BENPORATH_NOS_TARGETS in the NUPR1-high group (NES = 1.42, FDR = 0.00667), together with a positive enrichment trend for HALLMARK_GLYCOLYSIS (NES = 1.21, FDR = 0.136; Figure 1G,H). NUPR1 expression was positively correlated with SELM expression (Figure 1I). Together, these cohort-level associations provided a rationale for examining NUPR1-associated metabolic and stemness-related programs and for testing SP4 and SELM as candidate functional partners in bladder cancer cells.

### 3.2. NUPR1 Supports Viability, Glycolytic Activity, and Stemness-Associated Protein Expression in Bladder Cancer Cells

**Figure 2:**
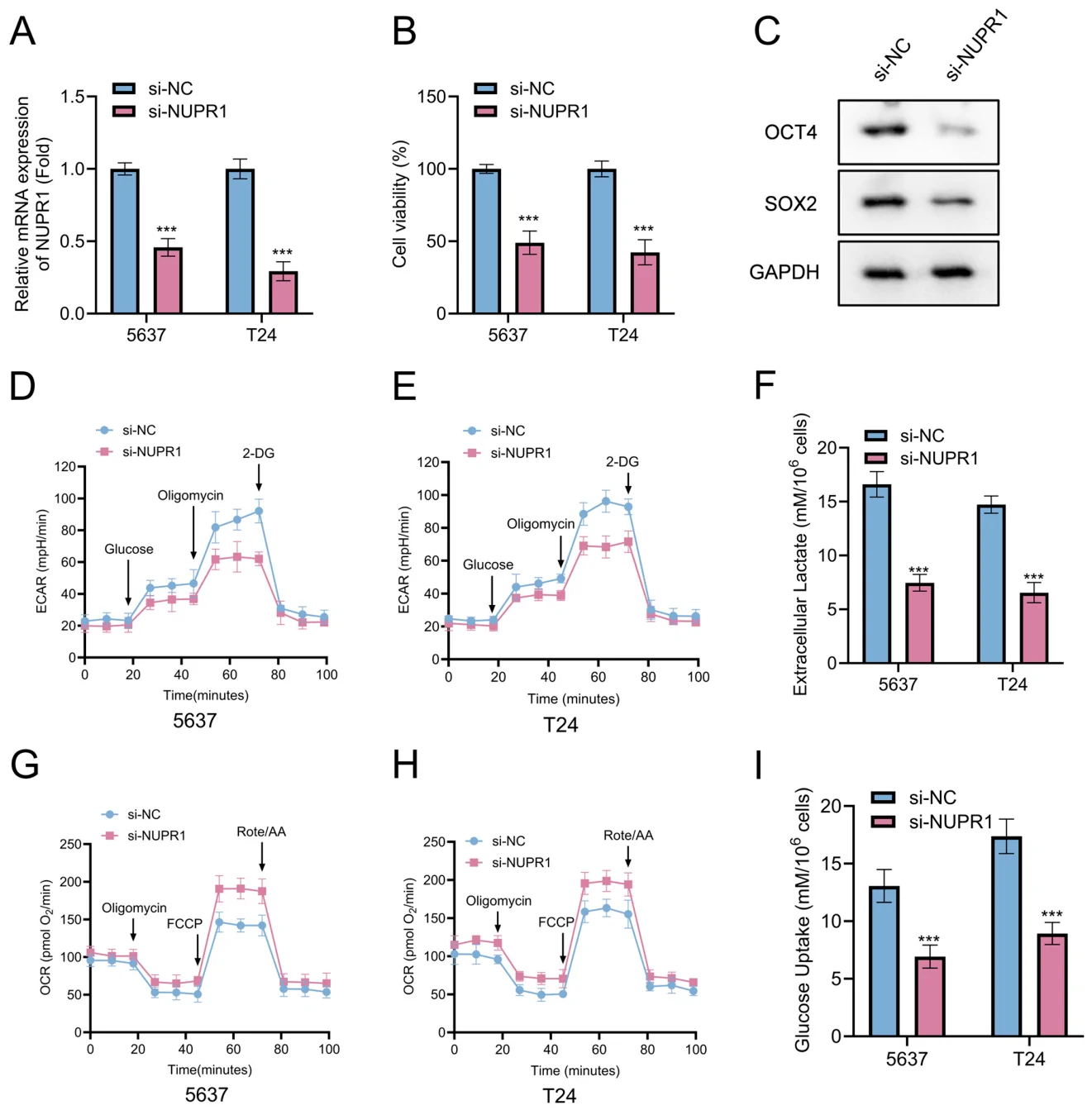
NUPR1 supports viability, glycolytic activity, and stemness-associated protein expression in bladder cancer cells. The whole blots (uncropped blots) are shown in Supplementary Figure S1. (**A**) RT-qPCR analysis of NUPR1 expression following si-NC or si-NUPR1 transfection. (**B**) CCK-8 measurements of cell viability. (**C**) Western blot analysis of OCT4 and SOX2, with GAPDH as the loading control. (**D**,**E**) ECAR profiles in 5637 and T24 cells. (**F**) Lactate production. (**G**,**H**) OCR profiles in 5637 and T24 cells. (**I**) Glucose uptake. Quantitative data are presented as mean ± SD from three independent experiments. *** *p* < 0.001 for the comparisons indicated in the panels.

NUPR1 was silenced in 5637 and T24 cells to examine its functional contribution. RT-qPCR confirmed reduced NUPR1 expression after transfection (Figure 2A), and CCK-8 assays showed decreased cell viability (Figure 2B). Western blotting demonstrated reduced OCT4 and SOX2 expression following NUPR1 depletion (Figure 2C), linking NUPR1 to the regulation of stemness-associated proteins.

NUPR1 depletion reduced ECAR in both cell lines (Figure 2D,E), accompanied by lower lactate production (Figure 2F) and glucose uptake (Figure 2I). OCR increased following NUPR1 depletion (Figure 2G,H), indicating a change in mitochondrial respiratory activity. Collectively, these results support a role for NUPR1 in maintaining glycolytic activity and stemness-associated protein expression in bladder cancer cells.

### 3.3. SP4 Interacts with NUPR1 and Participates in NUPR1 Promoter Regulation

**Figure 3:**
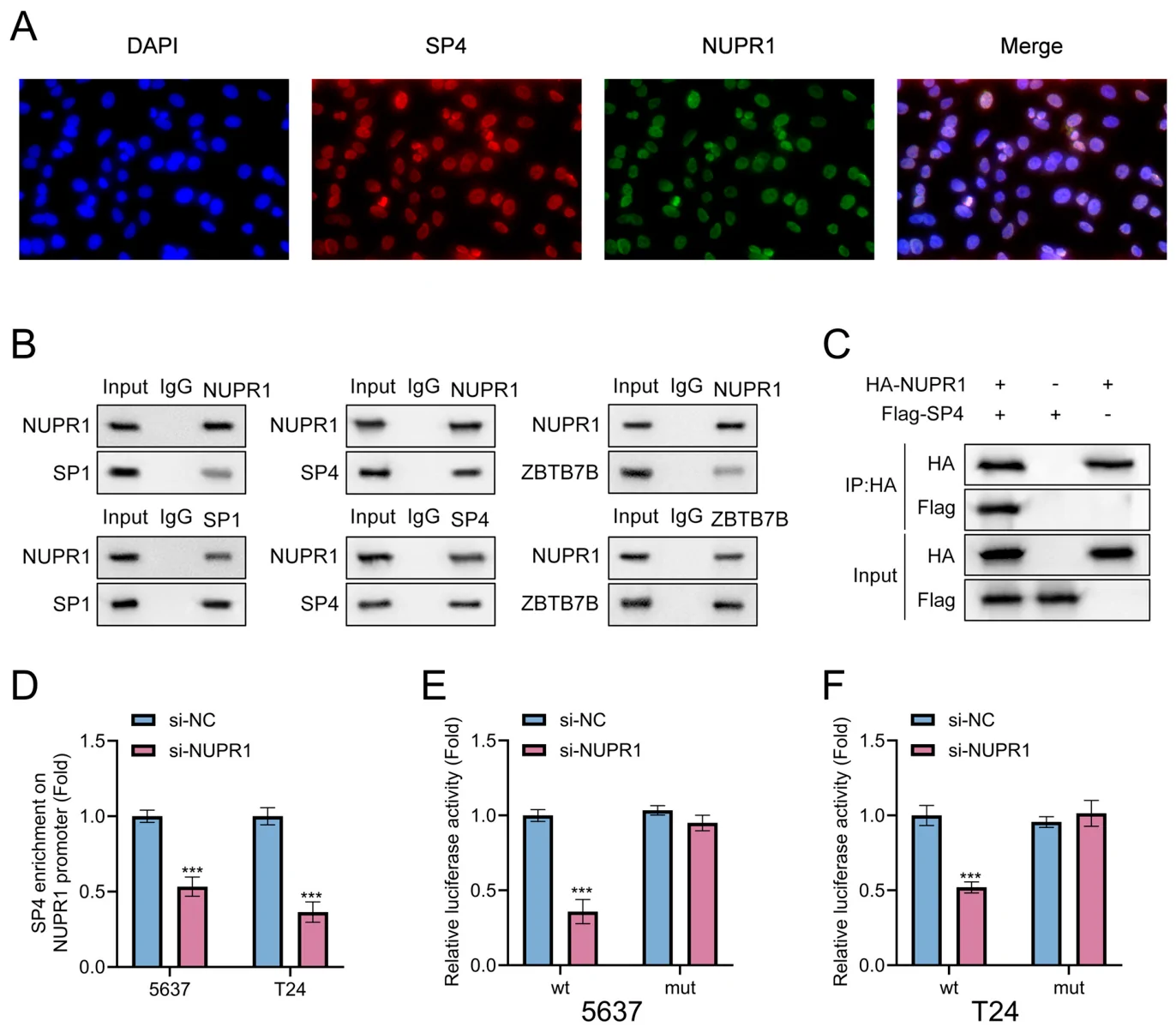
SP4 interacts with NUPR1 and participates in NUPR1 promoter regulation. (**A**) Immunofluorescence analysis of SP4 and NUPR1 localization, with DAPI nuclear counterstaining. (**B**) Endogenous co-immunoprecipitation examining associations of NUPR1 with SP1, SP4, and ZBTB7B. (**C**) Exogenous co-immunoprecipitation using HA-NUPR1 and Flag-SP4. (**D**) ChIP-qPCR analysis of SP4 enrichment at the NUPR1 promoter region following si-NC or si-NUPR1 transfection. (E,F) Relative activity of wild-type and mutant NUPR1 reporters in 5637 and T24 cells. Quantitative data are presented as mean ± SD from three independent experiments. *** *p* < 0.001 for the comparisons indicated in the panels.

Immunofluorescence showed overlapping nuclear localization of SP4 and NUPR1 (Figure 3A). Endogenous co-immunoprecipitation supported an association between SP4 and NUPR1, alongside the assessment of SP1 and ZBTB7B (Figure 3B). Exogenous co-immunoprecipitation using HA-NUPR1 and Flag-SP4 further supported the interaction (Figure 3C).

ChIP-qPCR detected SP4 enrichment at the assayed NUPR1 promoter region, which was reduced following NUPR1 depletion (Figure 3D). NUPR1 depletion reduced wild-type NUPR1 reporter activity in both cell lines, whereas the difference between si-NC and si-NUPR1 was not statistically significant for the mutant reporter (Figure 3E,F). Together, the interaction, ChIP-qPCR, and reporter findings are consistent with SP4-associated regulation of the NUPR1 promoter, with NUPR1 depletion affecting both SP4 enrichment and reporter activity.

### 3.4. SP4 Overexpression Partially Rescues the Effects of NUPR1 Depletion

**Figure 4:**
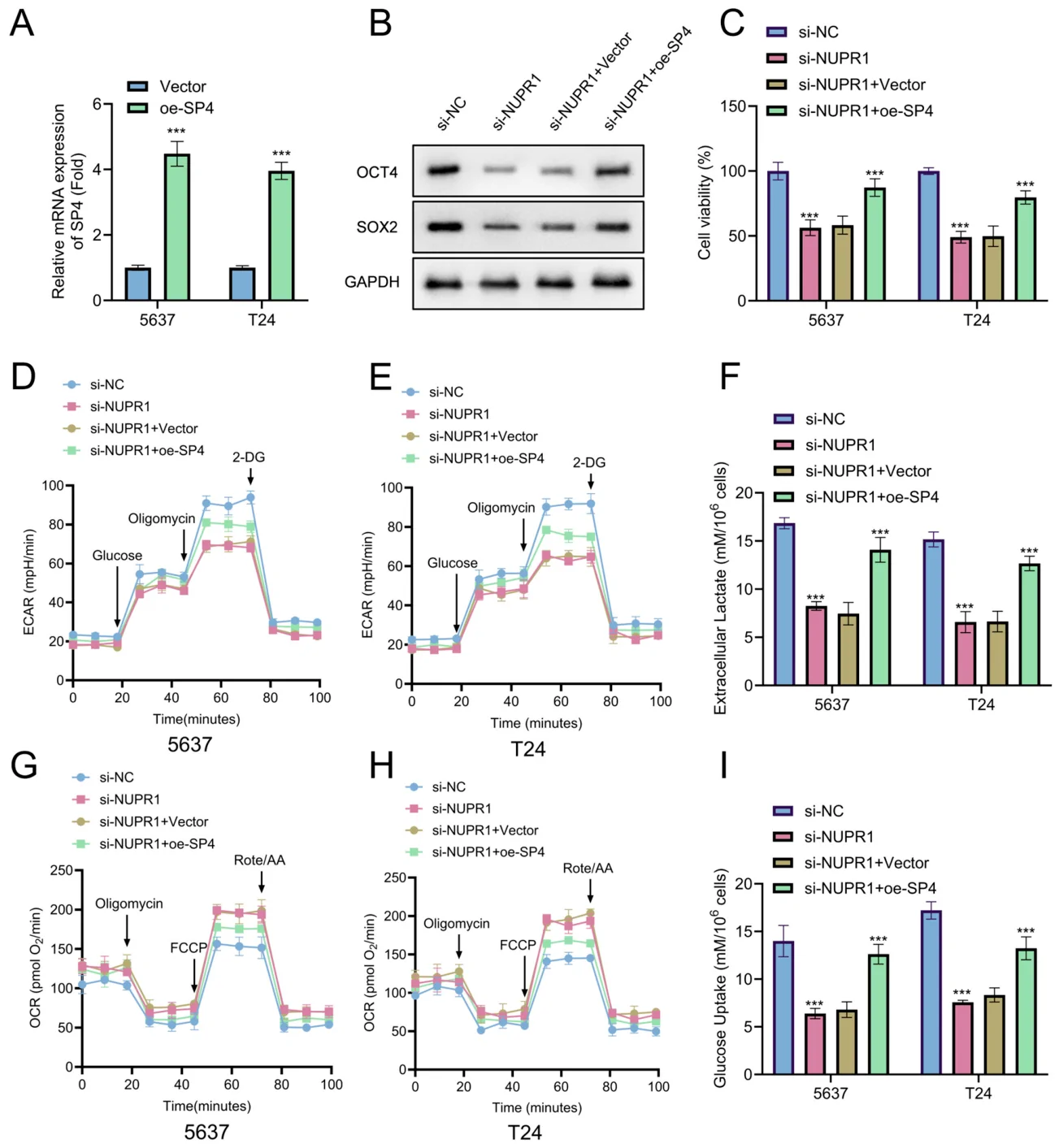
SP4 overexpression partially rescues the effects of NUPR1 depletion. (**A**) RT-qPCR confirmation of SP4 overexpression. (**B**) Western blot analysis of OCT4 and SOX2 in si-NC, si-NUPR1, si-NUPR1+Vector, and si-NUPR1+oe-SP4 groups, with GAPDH as the loading control. (**C**) CCK-8 measurements of cell viability. (**D**,**E**) ECAR profiles. (**F**) Lactate production. (**G**,**H**) OCR profiles. (**I**) Glucose uptake. Quantitative data are presented as mean ± SD from three independent experiments. *** *p* < 0.001 for the comparisons indicated in the panels.

To test whether increased SP4 expression could counteract the effects of NUPR1 depletion, SP4 was overexpressed in NUPR1-silenced cells. RT-qPCR confirmed increased SP4 expression (Figure 4A). Western blotting showed partial recovery of OCT4 and SOX2 expression following SP4 overexpression (Figure 4B). Cell viability was higher in the si-NUPR1+oe-SP4 group than in the si-NUPR1+Vector group (Figure 4C).

SP4 overexpression partially recovered ECAR (Figure 4D,E), lactate production (Figure 4F), and glucose uptake (Figure 4I). SP4 overexpression reduced the elevated OCR observed in NUPR1-depleted cells (Figure 4G,H). These results show that SP4 overexpression partially counteracts the metabolic and cellular effects of NUPR1 depletion, indicating functional overlap between SP4-associated and NUPR1-associated regulation.

### 3.5. NUPR1 Promotes SELM Expression, and SELM Contributes to NUPR1-Associated Metabolic and Cellular Phenotypes

**Figure 5:**
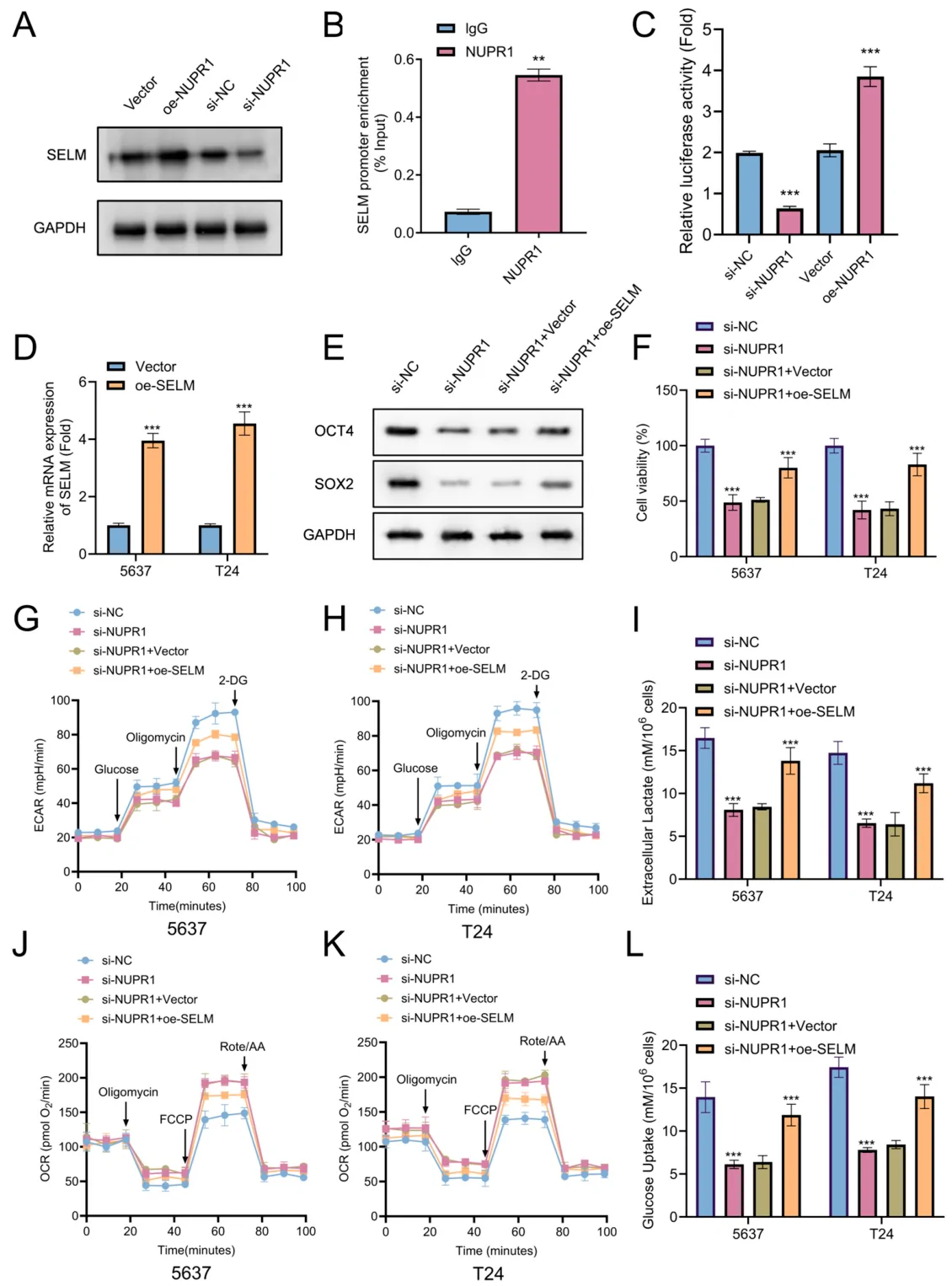
NUPR1 promotes SELM expression, and SELM partially rescues NUPR1-depletion-associated phenotypes. (**A**) Western blot analysis of SELM following NUPR1 overexpression or depletion, with GAPDH as the loading control. (**B**) ChIP-qPCR analysis of NUPR1 enrichment at the SELM promoter region. (**C**) Relative SELM reporter activity under the indicated conditions. (**D**) RT-qPCR confirmation of SELM overexpression. (**E**) Western blot analysis of OCT4 and SOX2 in si-NC, si-NUPR1, si-NUPR1+Vector, and si-NUPR1+oe-SELM groups. (**F**) CCK-8 measurements of viability. (**G**,**H**) ECAR profiles. (**I**) Lactate production. (**J**,**K**) OCR profiles. (**L**) Glucose uptake. Quantitative data are presented as mean ± SD from three independent experiments. ** *p* < 0.01 and *** *p* < 0.001 for the comparisons indicated in the panels.

NUPR1 overexpression increased SELM protein expression, whereas NUPR1 depletion reduced it (Figure 5A). ChIP-qPCR detected NUPR1 enrichment at the assayed SELM promoter region (Figure 5B), and reporter assays showed increased SELM reporter activity following NUPR1 overexpression (Figure 5C). These complementary observations support promoter-associated regulation of SELM by NUPR1.

To determine whether increasing SELM expression could compensate for NUPR1 depletion, SELM was overexpressed in NUPR1-silenced cells. RT-qPCR confirmed increased SELM expression (Figure 5D), and Western blotting showed partial recovery of OCT4 and SOX2 expression (Figure 5E). Cell viability was higher in the si-NUPR1+oe-SELM group than in the si-NUPR1+Vector group (Figure 5F). SELM overexpression also partially recovered ECAR (Figure 5G,H) and increased lactate production (Figure 5I) and glucose uptake (Figure 5L). SELM overexpression reduced the elevated OCR observed in NUPR1-depleted cells (Figure 5J,K). The partial recovery of metabolic measurements, viability, and OCT4/SOX2 expression supports a functional contribution of SELM to the phenotypes affected by NUPR1 depletion.

### 3.6. SELM Depletion Attenuates the Malignant Cellular Phenotypes Induced by NUPR1 Overexpression

**Figure 6:**
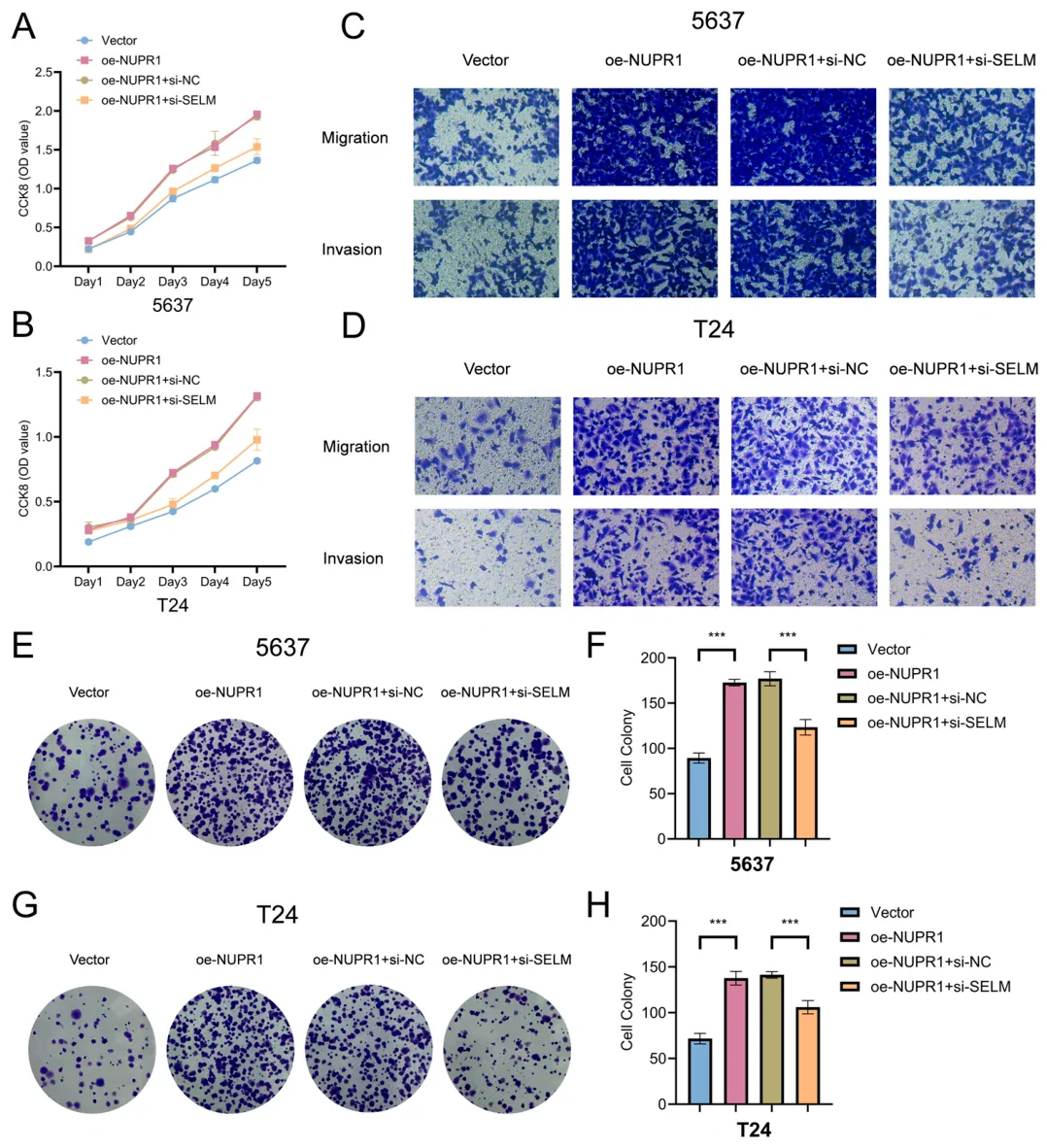
SELM depletion attenuates the malignant cellular phenotypes induced by NUPR1 overexpression (**A**,**B**) CCK-8 measurements in Vector, oe-NUPR1, oe-NUPR1+si-NC, and oe-NUPR1+si-SELM groups. (**C**,**D**) Representative Transwell migration and invasion images. (**E**,**G**) Representative colony formation images. (**F**,**H**) Quantification of colony numbers. Quantitative data are presented as mean ± SD from three independent experiments. *** *p* < 0.001 for the comparisons indicated in the panels.

We next examined whether SELM depletion could attenuate the effects of NUPR1 overexpression, providing a complementary test of the NUPR1–SELM functional relationship. CCK-8 assays showed increased viability following NUPR1 overexpression, which was attenuated by SELM depletion in both cell lines (Figure 6A,B). Transwell assays showed corresponding reductions in NUPR1-associated migration and invasion following SELM depletion (Figure 6C,D).

NUPR1 overexpression also increased colony formation, and this increase was attenuated by SELM depletion in both cell lines (Figure 6E,H). Together, these results support a contribution of SELM to NUPR1-associated viability, migration, invasion, and clonogenic growth.

### 3.7. NUPR1 Depletion Suppresses Xenograft Growth, with Partial Rescue by SP4 Overexpression

**Figure 7:**
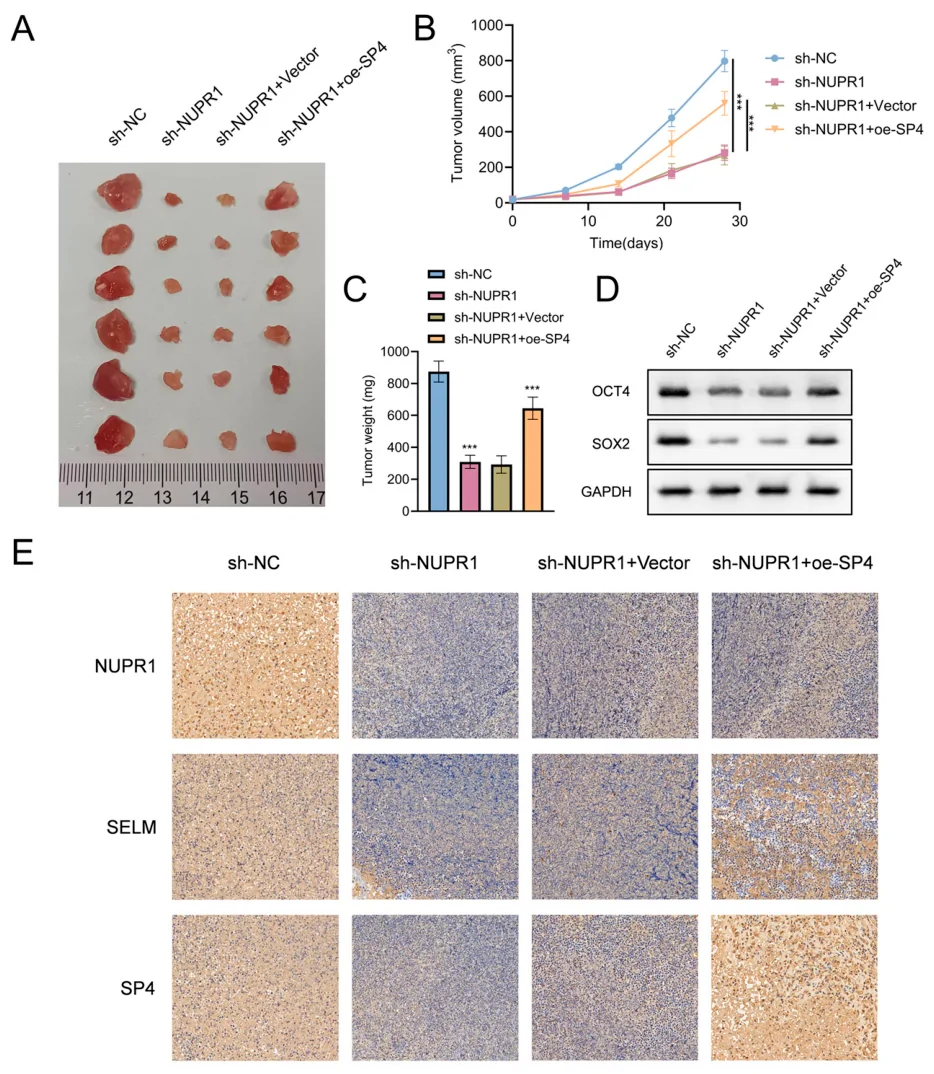
NUPR1 depletion suppresses xenograft growth, with partial rescue by SP4 overexpression. (**A**) Excised xenograft tumors from sh-NC, sh-NUPR1, sh-NUPR1+Vector, and sh-NUPR1+oe-SP4 groups. (**B**) Tumor-volume measurements during the experiment. (**C**) Tumor weights at the day-28 endpoint. (**D**) Western blot analysis of OCT4 and SOX2 in xenograft tissues. (**E**) Representative immunohistochemical staining of NUPR1, SELM, and SP4. Each animal group contained six mice. Tumor-volume and tumor-weight data are presented as mean ± SD. *** *p* < 0.001 for the comparisons indicated in the panels.

Xenograft experiments were performed using stable 5637 cells in four groups, with six mice per group. Tumors from the sh-NUPR1 group were smaller than those from the sh-NC group, while SP4 overexpression partially rescued tumor growth in the NUPR1-depleted background (Figure 7A). NUPR1 depletion reduced tumor volume and endpoint tumor weight, while SP4 overexpression partially reversed these reductions (Figure 7B,C).

Western blotting of xenograft tissues showed reduced OCT4 and SOX2 expression following NUPR1 depletion and partial recovery following SP4 overexpression (Figure 7D). Representative immunohistochemical images showed weaker SELM staining in the sh-NUPR1 group and partial recovery in the sh-NUPR1+oe-SP4 group (Figure 7E). These findings support an effect of NUPR1 depletion and SP4 overexpression on xenograft growth, accompanied by changes in OCT4/SOX2 expression and SELM staining.

## 4. Discussion

In the present study, we identified an SP4–NUPR1–SELM regulatory axis that links glycolytic activity with stemness-associated phenotypes in bladder cancer models. NUPR1 depletion reduced viability, glucose uptake, lactate production, ECAR, and OCT4/SOX2 expression. SP4 or SELM overexpression partially rescued these changes, while SELM depletion attenuated NUPR1-associated malignant cellular behaviors. NUPR1 depletion also suppressed xenograft growth, with partial rescue following SP4 overexpression. By integrating transcriptomic screening, interaction assays, promoter-associated analyses, and functional rescue experiments, this study connects NUPR1-associated transcriptional regulation with metabolic and cellular adaptation.

The metabolic findings highlight NUPR1 as a contributor to glycolytic activity in bladder cancer cells. Glycolysis supports tumor growth by supplying energy and intermediates for biosynthesis, while metabolic flexibility enables adaptation to changing cellular conditions [7,8,9,10,11,12,38]. The concordant reductions in ECAR, glucose uptake, and lactate production following NUPR1 depletion provide complementary evidence of altered glucose metabolism. The accompanying increase in OCR suggests a rebalancing of metabolic activity, consistent with the adaptive nature of cancer metabolism [9,10,11,12,13,39,40]. These observations extend the established stress-responsive functions of NUPR1 to glucose-related metabolic regulation in the tested bladder cancer models.

NUPR1-associated metabolic changes were accompanied by changes in OCT4 and SOX2 expression. These transcription factors participate in stemness-related programs and have been linked to aggressive tumor behavior [14,15,16,17,18,19,20]. Their reduction following NUPR1 depletion and partial recovery after SP4 or SELM overexpression indicate that the proposed network influences stemness-associated molecular features. Colony formation provided a separate measure of clonogenic growth. Together, these findings identify coordinated effects on glucose metabolism, stemness-associated proteins, and clonogenic behavior, providing a rationale for further examination of functional self-renewal.

The SP4 experiments provide insight into the transcription-associated regulation of NUPR1. SP-family proteins regulate multiple processes relevant to cancer growth and metabolism [27,28,29,30,31]. Endogenous and exogenous co-immunoprecipitation supported SP4–NUPR1 association, while ChIP-qPCR and reporter experiments implicated the assayed NUPR1 promoter region. SP4 overexpression also partially counteracted the effects of NUPR1 depletion. Such compensation may involve residual NUPR1 activity or additional SP4-regulated pathways. Accordingly, these findings support functional cooperation without requiring an exclusively linear SP4-to-NUPR1 relationship.

SELM emerged as a downstream functional component of this network. NUPR1 perturbation altered SELM protein expression, and the ChIP-qPCR and reporter findings supported promoter-associated regulation. The complementary rescue experiments extended this relationship beyond changes in expression: SELM overexpression partially counteracted the effects of NUPR1 depletion, while SELM depletion attenuated NUPR1-associated viability, migration, invasion, and colony formation. These findings identify SELM as a contributor to NUPR1-associated cellular effects and connect a selenoprotein-related homeostatic function with metabolic adaptation in bladder cancer.

The functional relationship between SELM and glucose metabolism may reflect its role in cellular homeostasis. Experimental studies in neural cell models have linked SELM to oxidative stress responses and cytosolic calcium regulation [41], while separate work has shown that mitochondrial ROS can participate in hypoxia-responsive transcription [42]. These findings identify redox- and calcium-sensitive signaling as plausible routes connecting SELM to metabolic regulation. In the present study, SELM overexpression partially recovered ECAR, glucose uptake, and lactate production after NUPR1 depletion, supporting a contribution to the measured metabolic phenotype. The relevant signaling intermediates and their effects on glucose transporters or glycolytic enzymes remain to be determined.

The xenograft findings further support the biological relevance of NUPR1-associated regulation. Reduced tumor growth following NUPR1 depletion and partial rescue by SP4 overexpression were accompanied by changes in OCT4/SOX2 expression and staining of the proteins involved in the proposed network. Together with the in vitro results, these observations provide a basis for investigating how stress-responsive transcriptional regulation and metabolic adaptation cooperate to support bladder cancer growth.

The findings should be interpreted within the scope of the experimental models. Functional assays were conducted in 5637 and T24 cells without low-grade/NMIBC or non-malignant urothelial comparators; therefore, the results concern regulation within the tested cancer models rather than the NMIBC-to-MIBC transition or tumor-selective activity. The TCGA associations were evaluated at the overall-cohort level without subtype- or stage-stratified analysis, and the inclusion of 409 tumor samples from 406 patients warrants patient-level sensitivity analysis. Independent patient specimens will be important for evaluating protein-level relationships and clinical relevance.

Further experiments are needed to resolve sequence-specific promoter regulation and the molecular intermediates connecting SELM to glycolytic activity. OCT4/SOX2 expression and adherent colony formation capture stemness-associated molecular features and clonogenic growth, while functional self-renewal remains to be examined. In vivo SELM rescue is also needed to extend the cell-based findings. The xenograft study focused on tumor growth following genetic manipulation of the implanted cancer cells. Longitudinal body-weight data are not reported, and dedicated studies will be needed to evaluate the systemic tolerability of pharmacological NUPR1 inhibition.

## 5. Conclusions

This study supports an SP4–NUPR1–SELM regulatory network associated with glycolytic activity and stemness-associated phenotypes in bladder cancer models. SP4 associates with NUPR1 and is implicated in NUPR1 promoter regulation, while NUPR1 promotes SELM expression. Complementary perturbation experiments support a functional contribution of SELM to NUPR1-associated metabolic and cellular effects. NUPR1 depletion also suppresses xenograft growth, with partial rescue following SP4 overexpression. These findings connect transcription-associated regulation with metabolic adaptation and identify functional relationships for further mechanistic and clinical investigation. 

## Data Availability

The public transcriptome data analyzed in this study are available from UCSC Xena/GDC TCGA-BLCA. Other data generated or analyzed during the current study are available from the corresponding author upon reasonable request.

## Supplementary Materials

The following supporting information can be downloaded at: https://www.mdpi.com/article/10.3390/cancers18203396/s1. Figure S1: Western blot source images corresponding to the indicated main-figure panels; Table S1: Primer sequences used for RT-qPCR; Table S2: siRNA duplex sequences and shRNA target sequences.

## Abbreviations

The following abbreviations are used in this manuscript:

NUPR1	nuclear protein 1
SP4	specificity protein 4
SELM	selenoprotein M
TCGA-BLCA	The Cancer Genome Atlas Bladder Cancer cohort
SP	specificity protein
TMM	trimmed mean of M-values
FDR	false discovery rate
MSigDB	Molecular Signatures Database
GSEA	gene set enrichment analysis
ssGSEA	single-sample gene set enrichment analysis
ChIP-qPCR	chromatin immunoprecipitation-quantitative polymerase chain reaction
mTORC1	mechanistic target of rapamycin complex 1
ECAR	extracellular acidification rate
OCT4	octamer-binding transcription factor 4
SOX2	SRY-box transcription factor 2
OCR	oxygen consumption rate
NMIBC	non-muscle-invasive bladder cancer
MIBC	muscle-invasive bladder cancer
ATP	adenosine triphosphate
SP1	specificity protein 1
RT-qPCR	reverse transcription-quantitative polymerase chain reaction
DMEM	Dulbecco’s modified Eagle’s medium
CCK-8	Cell Counting Kit-8
SD	standard deviation
ANOVA	analysis of variance
ROS	reactive oxygen species
