# Supplementary material for: The SP4–NUPR1–SELM Axis Promotes Glycolytic Reprogramming and Stemness-Associated Phenotypes in Bladder Cancer
Source: Cancers (Basel). 2026 Oct 20;18(20):3396. doi: 10.3390/cancers18203396 (PMC13702499; doi:10.3390/cancers18203396)
Supplement: Supplementary file 1 [file cancers-18-03396-s001.zip › Supplementary Files/Figure S1. Western blot source images corresponding to the indicated main-figure panels.pdf]

Figure2C OCT4

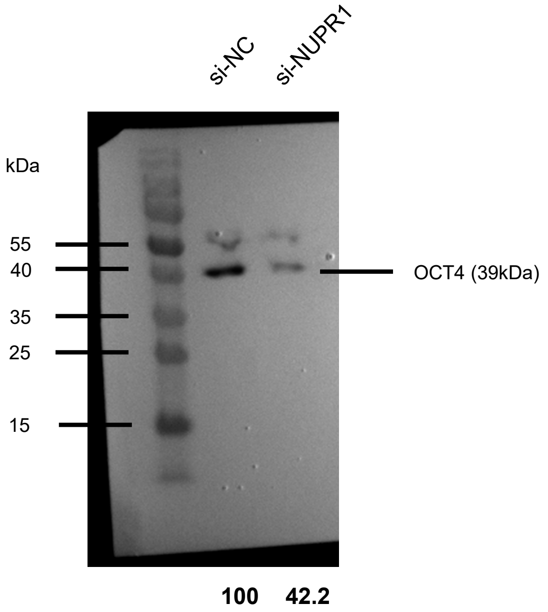

Figure2C SOX2

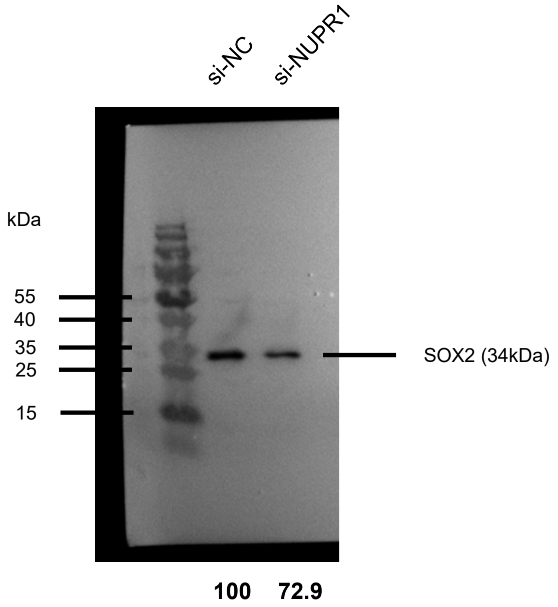

Figure2C GAPDH

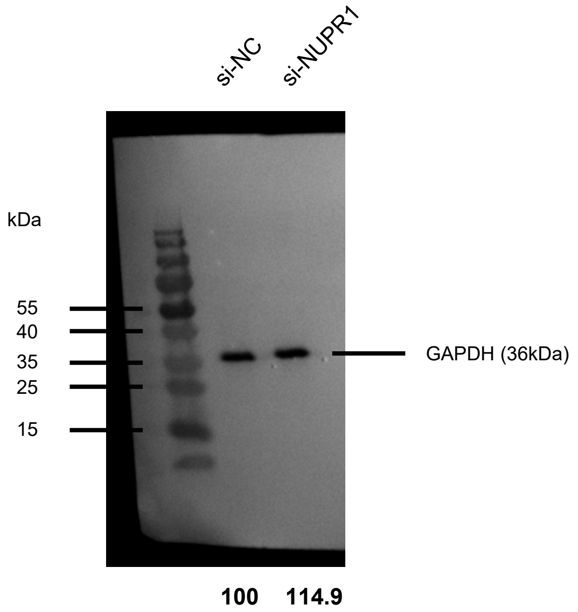

Figure3B NUPR1(1)

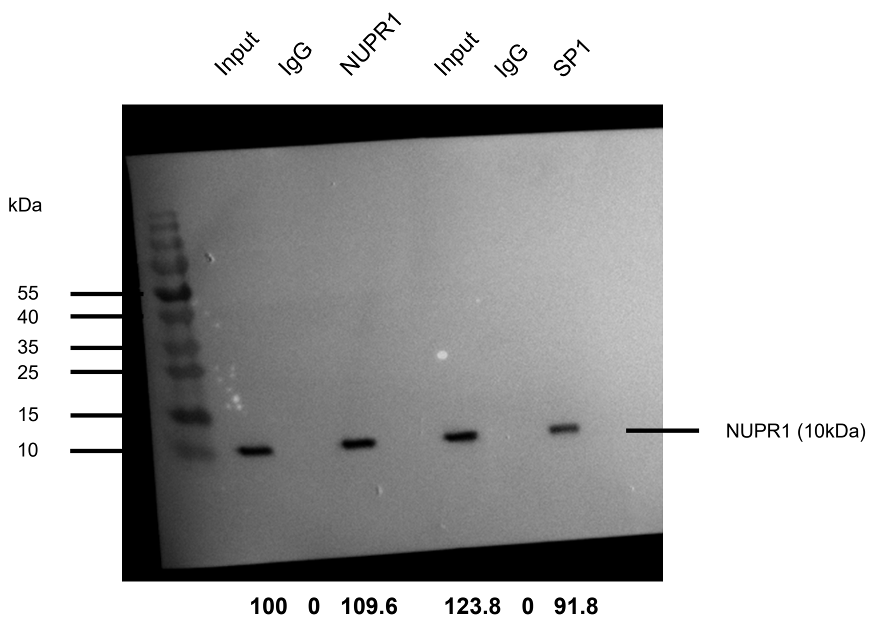

Figure3B NUPR1(2)

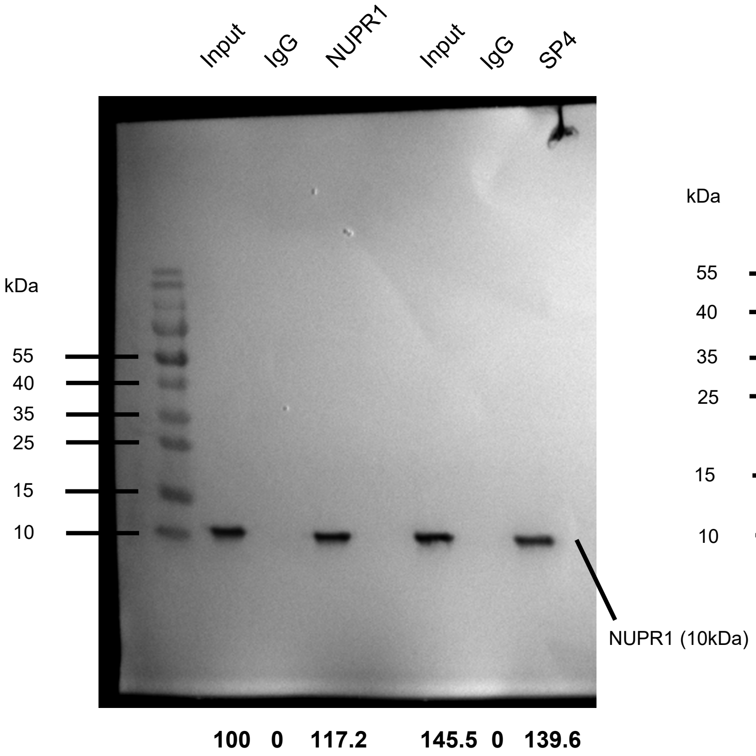

Figure3B NUPR1(3)

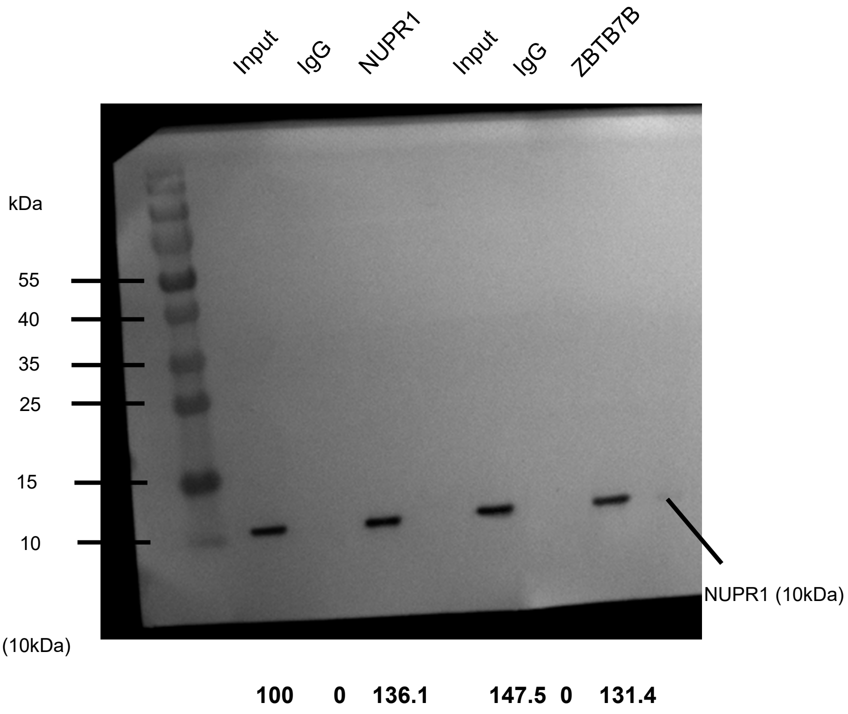

Figure3B SP1

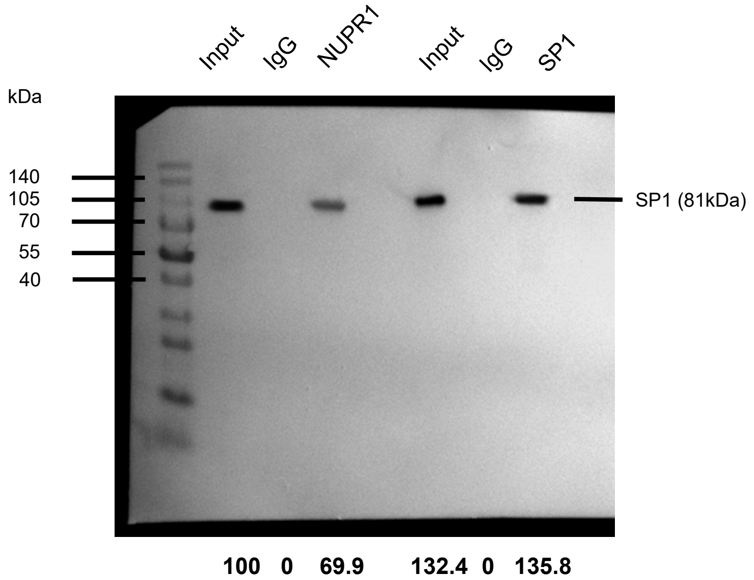

Figure3B SP4

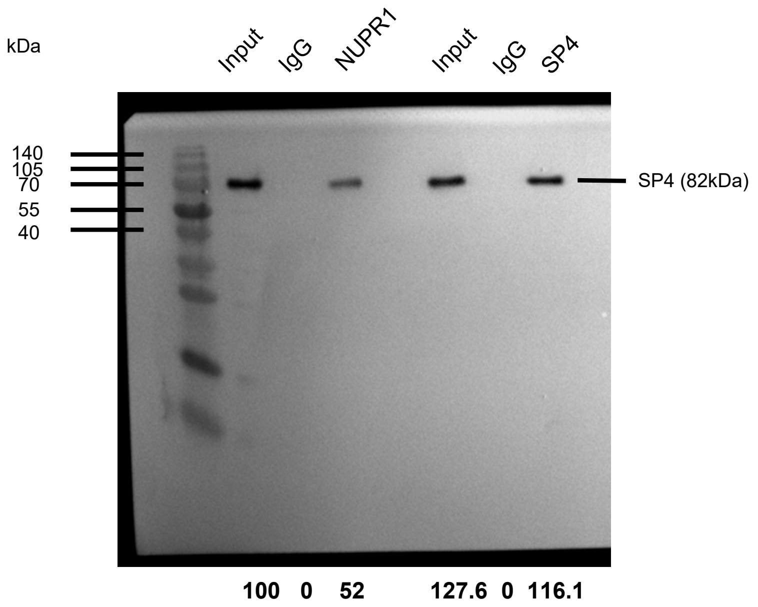

Figure3B ZBTB7B

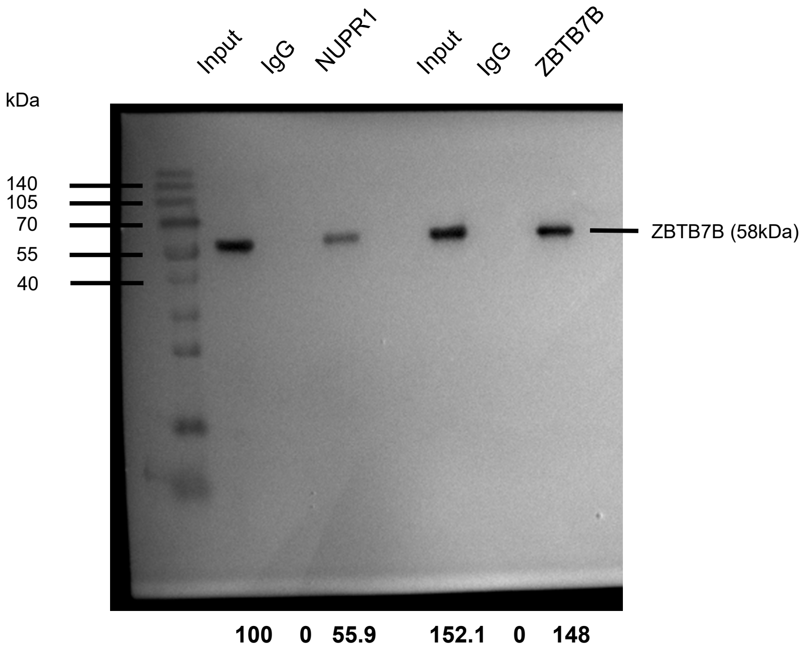

Figure3C IP:HA HA-NUPR1

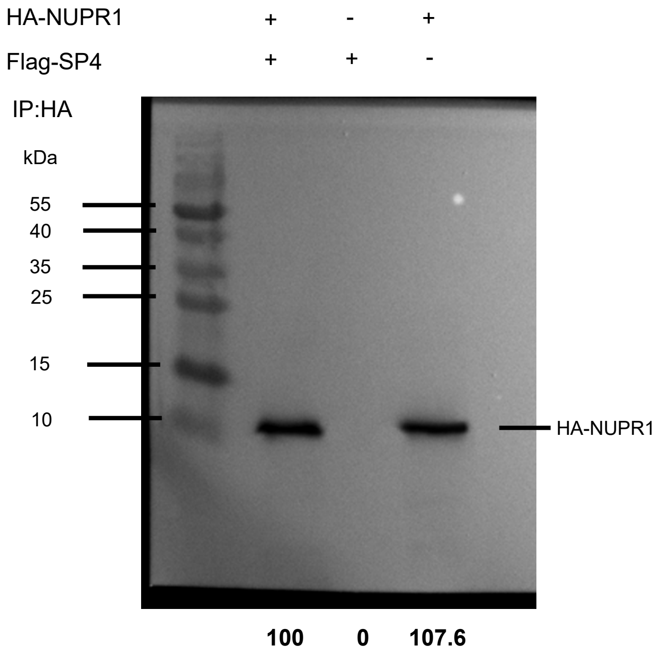

Figure3C IP:HA Flag-SP4

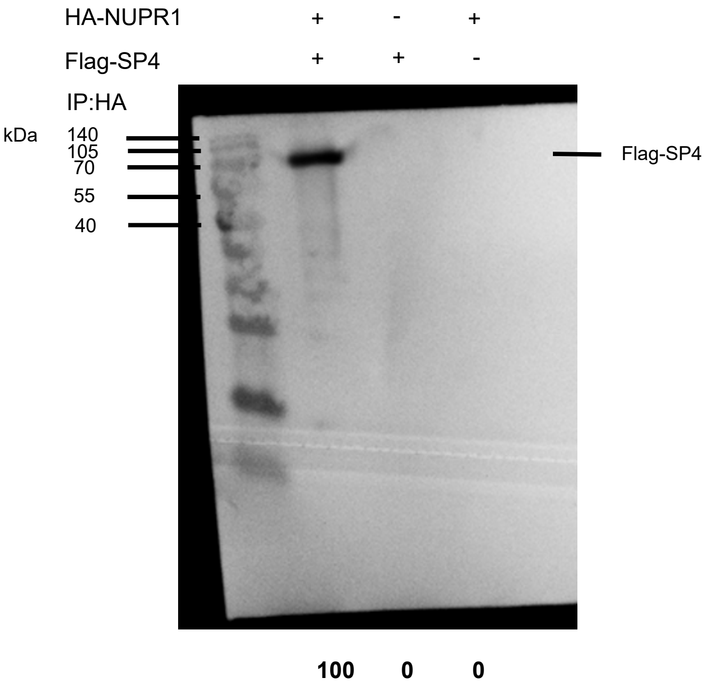

Figure3C Input HA-NUPR1

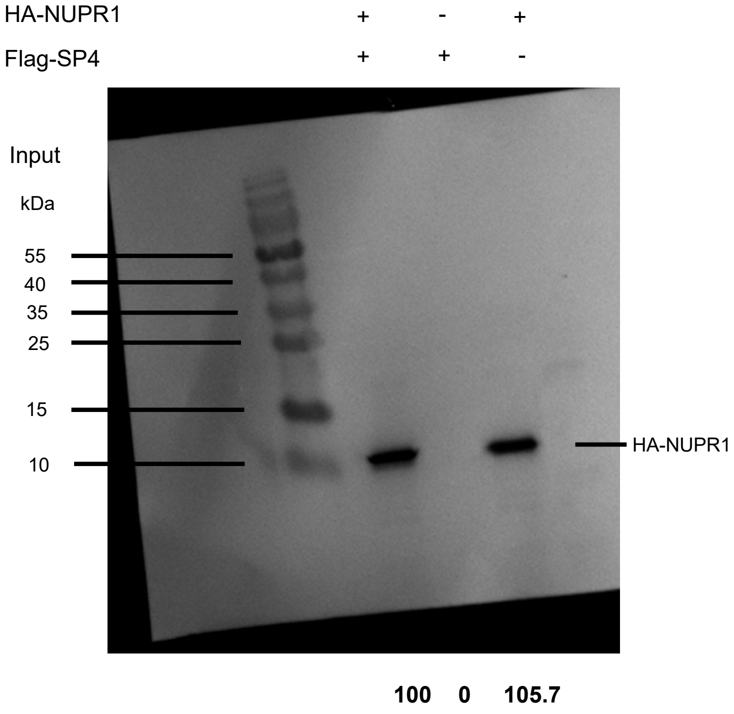

Figure3C Input Flag-SP4

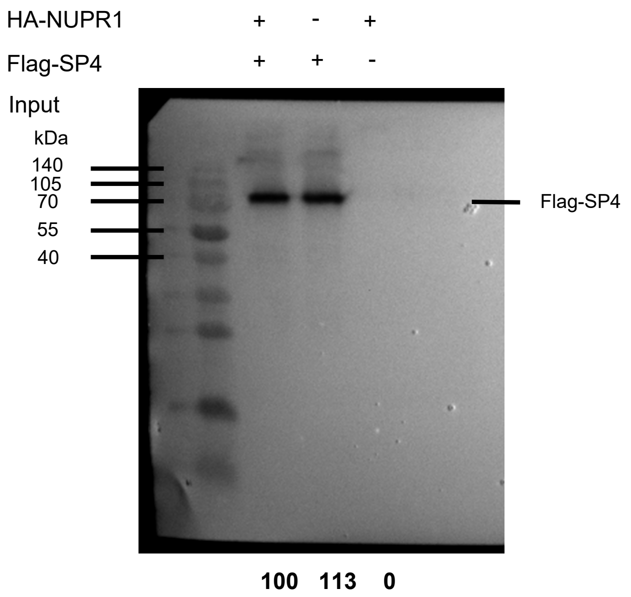

Figure4B OCT4

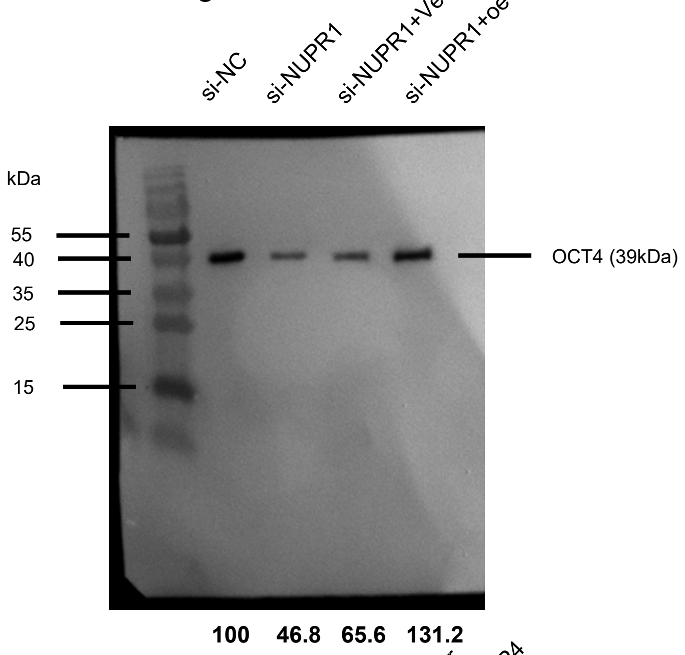

Figure4B SOX2

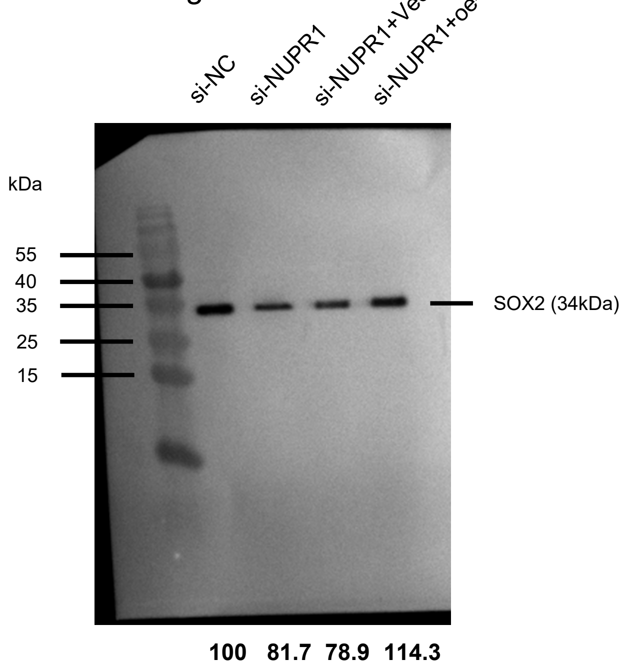

Figure4B GAPDH

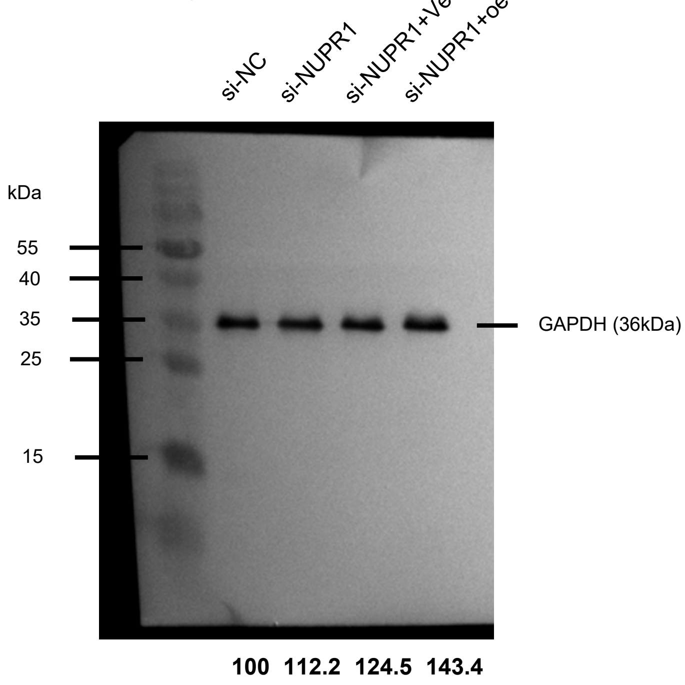

Figure5A SELM

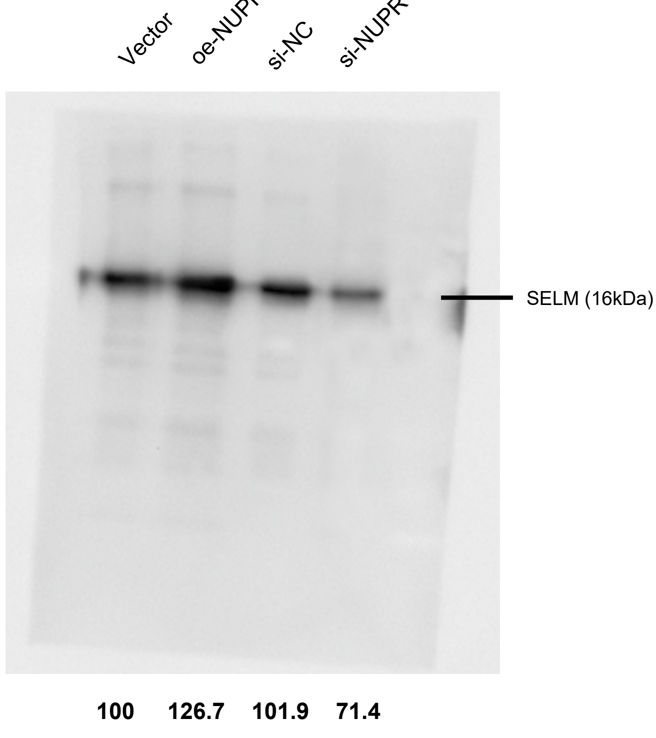

Figure5A GAPDH

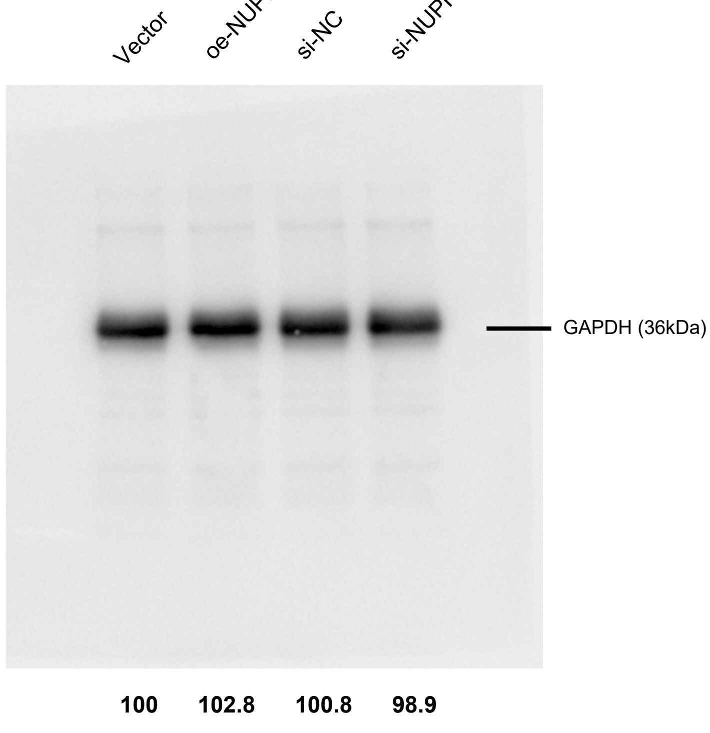

Figure5E OCT4

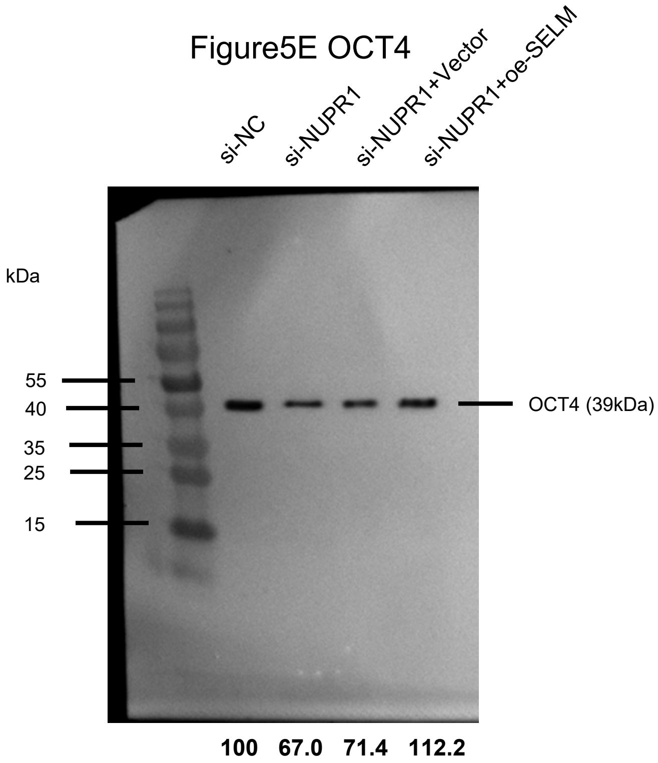

Figure5E SOX2

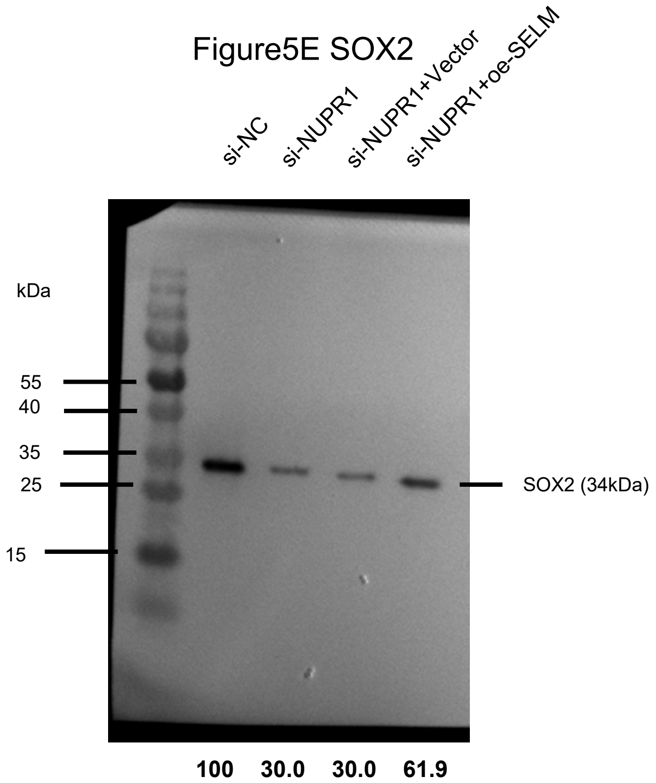

Figure5E GAPDH

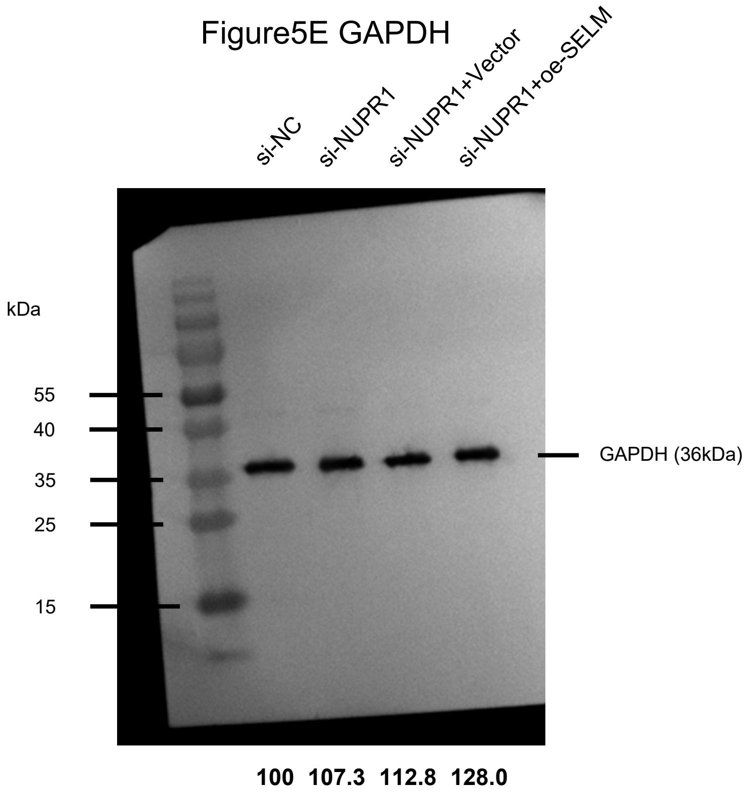

Figure7D OCT4

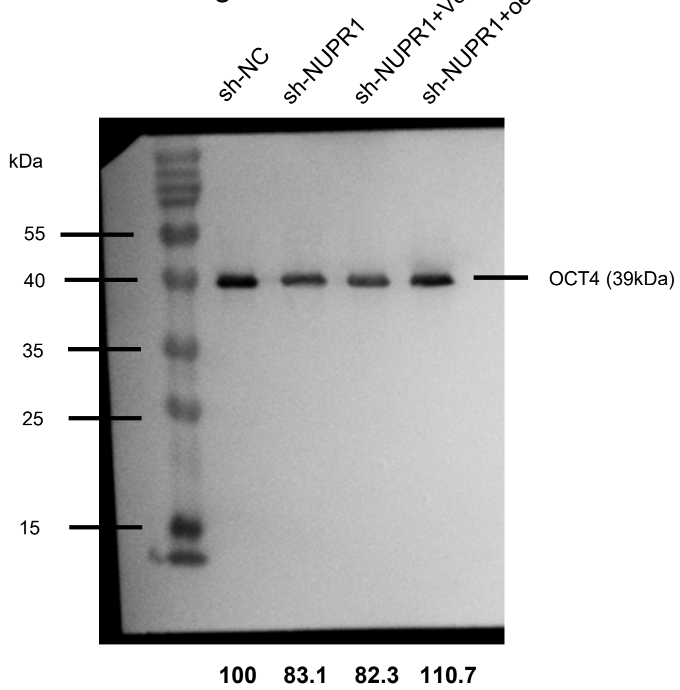

Figure7D SOX2

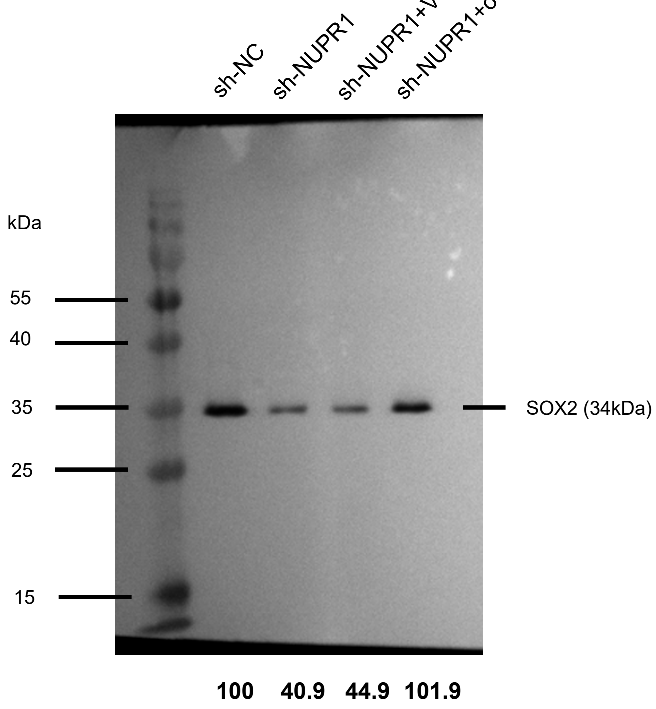

Figure7D GAPDH

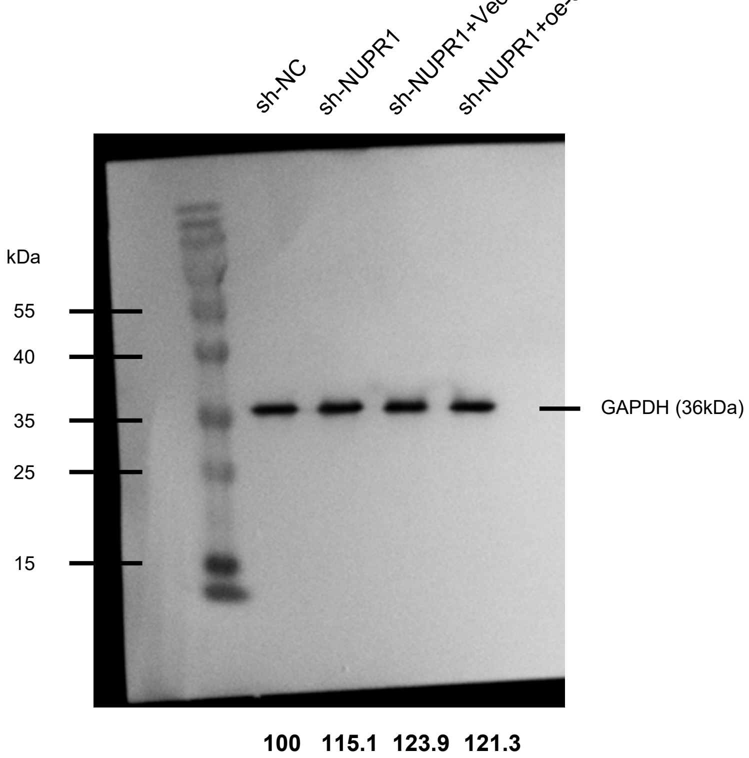

Relative band intensities (%) are shown below the corresponding lanes. Band intensities were quantified by densitometry with local background subtraction. Within each blot panel, the background-corrected integrated intensity of each band was divided by that of the leftmost sample lane and multiplied by 100, setting the leftmost sample lane to 100%.
